# Genome wide DNA methylation analysis of alveolar capillary dysplasia lung tissue reveals aberrant methylation of genes involved in development including the *FOXF1* locus

**DOI:** 10.1186/s13148-021-01134-1

**Published:** 2021-07-29

**Authors:** Evelien Slot, Ruben Boers, Joachim Boers, Wilfred F. J. van IJcken, Dick Tibboel, Joost Gribnau, Robbert Rottier, Annelies de Klein

**Affiliations:** 1 Department of Paediatric Surgery, Erasmus MC – Sophia Children’s Hospital Rotterdam, Rotterdam, Netherlands; 2 Department of Clinical Genetics, Rm Ee2089, Erasmus MC Rotterdam, Wytemaweg 80, 3015 CN Rotterdam, Netherlands; 3 Department of Developmental Biology, Oncode Institute, Erasmus MC Rotterdam, Rotterdam, Netherlands; 4 Center for Biomics, Erasmus University Medical Center, Erasmus MC, Rotterdam, Netherlands; 5 Department of Cell Biology, Erasmus University Medical Center, Erasmus MC, Rotterdam, Netherlands

**Keywords:** DNA methylation, ACD/MPV, Lung development, FOXF1

## Abstract

**Background:**

Alveolar capillary dysplasia with or without misalignment of the pulmonary veins (ACD/MPV) is a lethal congenital lung disorder associated with a variety of heterozygous genomic alterations in the *FOXF1* gene or its 60 kb enhancer. Cases without a genomic alteration in the *FOXF1* locus have been described as well. The mechanisms responsible for *FOXF1* haploinsufficiency and the cause of ACD/MPV in patients without a genomic *FOXF1* variant are poorly understood, complicating the search for potential therapeutic targets for ACD/MPV. To investigate the contribution of aberrant DNA methylation, genome wide methylation patterns of ACD/MPV lung tissues were compared with methylation patterns of control lung tissues using the recently developed technique Methylated DNA sequencing (MeD-seq).

**Results:**

Eight ACD/MPV lung tissue samples and three control samples were sequenced and their mutual comparison resulted in identification of 319 differentially methylated regions (DMRs) genome wide, involving 115 protein coding genes. The potentially upregulated genes were significantly enriched in developmental signalling pathways, whereas potentially downregulated genes were mainly enriched in O-linked glycosylation. In patients with a large maternal deletion encompassing the 60 kb *FOXF1* enhancer, DNA methylation patterns in this *FOXF1* enhancer were not significantly different compared to controls. However, two hypermethylated regions were detected in the 60 kb *FOXF1* enhancer of patients harbouring a *FOXF1* point mutation. Lastly, a large hypermethylated region overlapping the first *FOXF1* exon was found in one of the ACD/MPV patients without a known pathogenic *FOXF1* variation.

**Conclusion:**

This is the first study providing genome wide methylation data on lung tissue of ACD/MPV patients. DNA methylation analyses in the *FOXF1* locus excludes maternal imprinting of the 60 kb *FOXF1* enhancer. Hypermethylation at the 60 kb *FOXF1* enhancer might contribute to *FOXF1* haploinsufficiency caused by heterozygous mutations in the *FOXF1* coding region. Interestingly, DNA methylation analyses of patients without a genomic *FOXF1* variant suggest that abnormal hypermethylation of exon 1 might play a role in some ACD/MPV in patients.

**Supplementary Information:**

The online version contains supplementary material available at 10.1186/s13148-021-01134-1.

## Background

Alveolar capillary dysplasia with or without misalignment of the pulmonary veins (ACD/MPV) is a lethal congenital lung disorder associated with heterozygous genomic alterations in the Forkhead Box F1 (*FOXF1)* gene locus. These genomic alterations vary from point mutations to large copy number variations (CNVs) but all seem to cause haploinsufficiency leading to ACD/MPV [1, 2]. The molecular mechanism responsible for the haploinsufficiency are poorly understood which complicates the search for potential therapeutic targets for ACD/MPV. Furthermore, in approximately 30% of ACD/MPV patients no genomic variants in the *FOXF1* locus could be found, challenging the diagnostic process [3, 4].

The majority of ACD/MPV patients die within the first weeks of life due to insufficient gas exchange and therapy-resistant pulmonary hypertension. Histologically, ACD/MPV is characterized by reduced numbers of pulmonary capillaries, increased medial wall thickening in pulmonary arterioles and, in most cases, displaced pulmonary veins (reviewed in Slot et al. [2]). Genomic alterations associated with ACD/MPV are either small indel mutations in the *FOXF1* gene or large deletions that include the 60 kb enhancer region [4–6]. This enhancer is located 250 kb upstream of the *FOXF1* transcription start site and lies within the same topologically associated domain (TAD) as *FOXF1* [4]*.* Furthermore, this *FOXF1* enhancer region contains GLI2 binding sites that upregulate *FOXF1* expression upon Sonic hedgehog activation [4, 5, 7].

Heterozygous *FOXF1* variants seem to have a dominant negative effect that cause haploinsufficiency of *FOXF1*, associated with ACD/MPV, and it has been suggested that *FOXF1* is subjected to parental imprinting [1, 8–10]. One of the well-established mechanisms of parental imprinting is allele specific DNA methylation of gene promotors [11]. However, several studies have shown that the *FOXF1* promoter is not methylated in both normal and ACD/MPV lung tissue, excluding the possibility of allele specific silencing of *FOXF1* through promotor methylation [10, 12, 13]. Furthermore, *FOXF1* seems to be similarly transcribed from both alleles, which opposes imprinting of the *FOXF1* transcription region itself [13, 14]. However, this does not completely exclude the 60 kb enhancer region to be subject to parental imprinting on the paternal allele (Additional file 3: Figure S 1), as suggested previously [4] based on the finding that all but one of the large deletions detected in ACD/MPV patients involved the 60 kb enhancer on the maternal chromosome [3, 10]. Furthermore, although very unlikely, parental imprinting of the *FOXF1* enhancer on the maternal allele has never been excluded either. Up to now, studies investigating allele specific methylation in the 60 kb enhancer are limited and mainly use DNA isolated from blood or skin tissue which might not be representative for methylation patterns in lung tissue [15, 16].

Although most patients have a similar clinical course, atypical cases have been described that deviate in time of disease presentation and progression or reliance on life supportive care. So far, the phenotypical differences could not be correlated with the presence, type or location of the *FOXF1* variant. Considering the complex genotype–phenotype correlation and the absence of genomic *FOXF1* alterations in some of the patients, abnormal DNA methylation might contribute to the pathogenesis of ACD/MPV.

In 2018, we developed Methylated DNA Sequencing (MeD-seq) which enables genome wide DNA-methylation profiling at single-nucleotide resolution without the need for deep sequencing [17]. Using this technique, this study aimed at detection of genome wide methylation patterns in lung tissue of eight ACD/MPV patients, with specific focus on the *FOXF1* locus, to identify potential differences that might play a role in the pathogenesis of ACD/MPV.

## Results

### Differential methylation of developmental genes in ACD/MPV lung tissue

Formalin-fixed and paraffin-embedded (FFPE) lung tissue samples of eight ACD/MPV patients and three age-matched controls were available to study lung specific methylation (Table 1). All ACD/MPV patients have been previously described and tested for *FOXF1* alterations extensively [3]. Three patients with a point mutation in the first *FOXF1* exon (ACD-mut), three patients with a large deletion overlapping the *FOXF1* enhancer on the maternal allele (ACD-del) and two patients without a known pathogenic variant in the *FOXF1* locus (ACD-none) (Additional file 4: FigureS 2) were selected. Control patients were age-matched individuals with a medical condition unrelated to lung development or underlying pulmonary pathology.

**Table 1:** Overview of included ACD/MPV patients and control samples

Sample ID	FOXF1 alteration (Hg38)	Time of biopsy	Cause of death	Co-malformation
ACD-del1	Loss chr16: 86,103,904–86,253,076	Post mortem	ACD/MPV	None
ACD-del2	Loss chr16: 86,103,904–86,305,560	Post mortem	ACD/MPV	Omphalecele, hydronephrosis
ACD-del3	Loss chr16: 86,209,574–87,669,623	6 days of life	ACD/MPV	Chylothorax
ACD-mut1	chr16: 86510735C > G p.(L56V)	34 days of life	ACD/MPV	Hirshprung (clinical diagnosis)
ACD-mut2	chr16: 86510822T > A p.(F85I)	Post mortem	ACD/MPV	Atrial septal defect, ventricle septal defect, gall bladder agenesis, duodenal atresia, anal atresia, intestinal malrotation
ACD-mut3	chr16: 86510730delT p.(I54Tfs*16)	9 days of life	ACD/MPV	None
ACD-none1	None	Post mortem	ACD/MPV	None
ACD-none2	None*	Post mortem	ACD/MPV	None
C1	–	Post mortem	Ventriculomegaly	None
C2	–	Post mortem	Hypovolemic shock	None
C3	–	Post mortem	Asphyction	None

All ACD/MPV patients developed critical and life -threatening respiratory insufficiency within the first 24 h after birth

^*^This patient carried a duplication in the 3’UTR of *FOXF1* that was classified as likely benign according to the ACMG classification system [3]

**Fig. 1:**
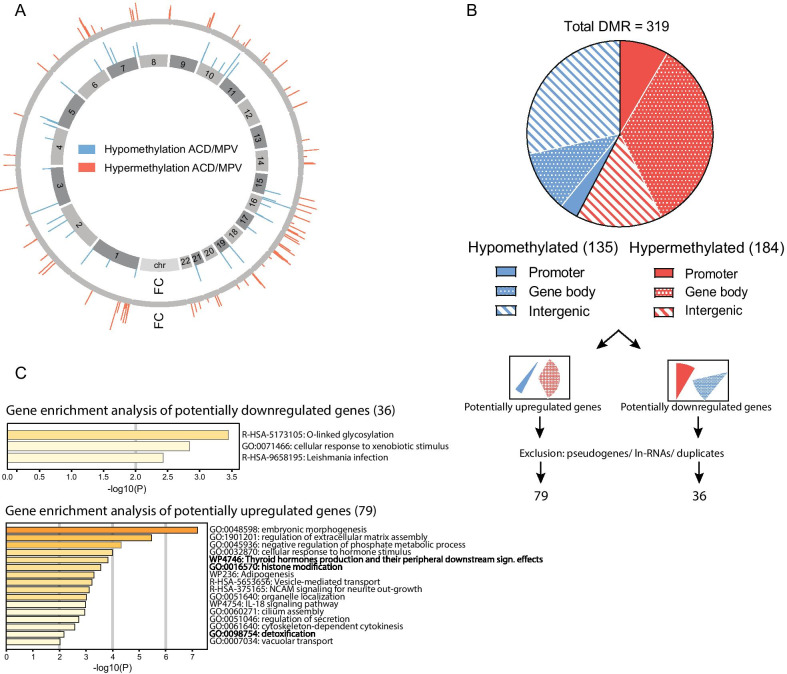
Genome wide methylation patterns of ACD/MPV lung tissues suggest abnormal gene regulation. **a** Hypermethylated (red) and hypomethylated (blue) regions in ACD/MPV lungs that were found genome wide (X- and Y-chromosome excluded; fold change < 2 excluded). The bar height indicates the relative fold change (FC) of the differentially methylated regions (DMRs). **b** Pie chart of the distribution of hyper- (red) and hypomethylated (blue) DMRs. **c** Results of gene ontology cluster enrichment analyses with Metascape [26] using the lists of genes that are potentially up- and downregulated based on the overlap of DMRs with promoters and gene bodies (squares with pie slices on the left indicate DMR overlap) (Additional file 1: table S 1)

Genome wide DNA methylation patterns of ACD/MPV lung tissues were compared with methylation patterns of control lung tissues. Differentially methylated regions (DMRs) located on the X- and Y-chromosome were removed because samples were not gender-matched, as well as DMRs with a fold change below 2. This resulted in 319 DMRs of which 184 were hypermethylated and 135 were hypomethylated in ACD/MPV lung samples (Fig. 1a). Approximately half of the DMRs (43%) were intergenic, the other 57% of DMRs fully or partly overlapped putative gene promoters (TSS) or gene bodies (Fig. 1b). Depending on the location, methylation acts differently on gene regulation. In general, promotor methylation is associated with gene silencing, whereas gene body methylation is mostly associated with gene activation [18–25]. Based on these assumptions, we labelled genes as potentially up- or downregulated in ACD/MPV lung tissue compared to control lung tissue. Exclusion of pseudogenes, long-non coding RNAs and duplicates resulted in 79 potentially upregulated and 36 potentially downregulated protein encoding genes (Fig. 1b, Additional file 1: table S 1)**.** To study if these potentially up- and downregulated genes are specifically involved in certain biological pathways, gene enrichment analyses were performed using Metascape [26]. Gene enrichment analysis of the potentially upregulated genes revealed 16 statistically significant enriched gene clusters of which the top cluster (named ‘embryonic morphogenesis’ (GO:0,048,598)) (Fig. 1c) included different signalling pathways involved in developmental processes such as ‘embryonic organ morphogenesis’ (GO:0,048,562) and ‘blood vessel morphogenesis’ (GO: 0,048,514) (Additional file 2: table S 2). The genes included in this top cluster were: *COL4A2, GATA2, HOXA3, HOXB3, HOXB6, HOXD3, NOTCH1, TGFB1, SOCS3, KDM6B, TENM4, CCDC40, TIE1, NXN, DHRS3, RXRA.* Gene enrichment analysis of potentially downregulated genes revealed three significantly overrepresented gene clusters, with the most significant pathway being ‘O-linked glycosylation’ which included *MUC5AC, ADAMTS2* and *GALNT15.*

### The FOXF1 enhancer is not maternally imprinted

**Fig. 2:**
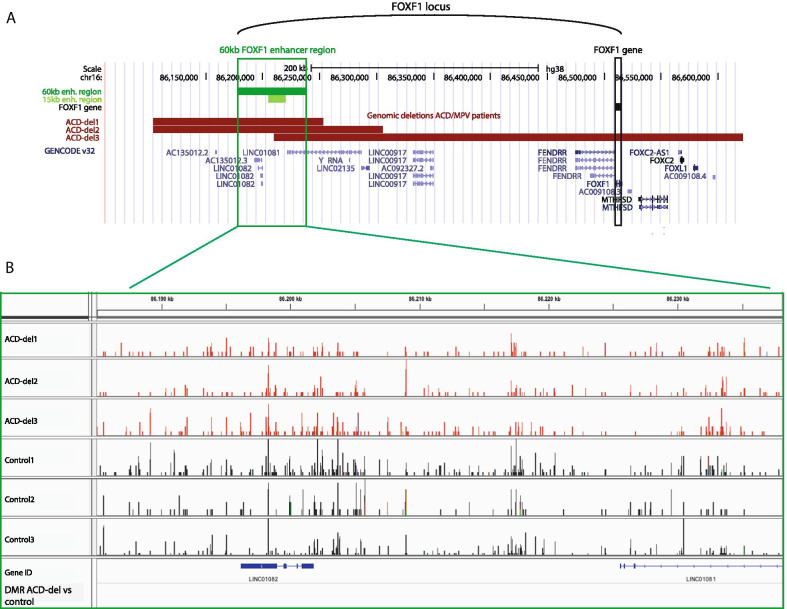
Absence of DMRs in ACD-del samples excludes maternal imprinting. **a** Overview of the *FOXF1* locus in the UCSC genome browser (GRCh38). The three maternal deletions of ACD-del samples involving the 60 kb *FOXF1* enhancer are depicted in red. **b** Methylation patterns of the three ACD-del (red) and control samples (grey) in the 60 kb *FOXF1* enhancer depicted in IGV viewer. No significant DMRs were detected in the *FOXF1* enhancer as shown by the empty ‘DMR ACD-del vs. control’ track

Next, we focused on methylation patterns in the *FOXF1* locus (Fig. 2a). Comparison of all ACD/MPV samples with controls revealed no significant DMRs in the *FOXF1* gene, the 60 kb *FOXF1* enhancer nor the 250 kb between these two regions (Table 2). In order to identify potential correlations between methylation patterns and patient-specific *FOXF1* variants, we separated the ACD/MPV samples with known genomic *FOXF1* variants into two groups. The first group (ACD-del) contained the three samples with a large deletion involving the *FOXF1* enhancer on the maternal chromosome (Fig. 2a), the second group (ACD-mut) contained the three ACD/MPV patients with a point mutation in the first exon of *FOXF1* (ACD-mut). Both groups were compared with control samples and analysed for significant DMRs in the *FOXF1* locus. In addition, we separately compared DNA methylation patterns of the two ACD/MPV patients without a pathogenic *FOXF1* variant (ACD-none1 and ACD-none2)[3] with control samples.

**Table 2:** DMRs in the *FOXF1* locus detected by statistical group comparisons

Group comparison	60 kb enhancer region	Between enhancer and promoter	FOXF1 promoter	FOXF1 gene body
ACD/MPV vs. Control	–	–	–	–
ACD-del vs. Control	–	–	–	–
ACD-mut vs. Control	chr16: 86,210,617–86,211,669 (FC 3.5)	chr16: 86,243,281–86,243,394 (FC 1.1)	–	–
	chr16: 86,212,910–86,213,514 (FC 2.7)	chr16: 86,345,534–86,345,640 (FC1.4)		
		chr16: 86,504,259–86,504,462 (FC1.1)		
		chr16: 86,504,711–86,505,743 (FC 7.3)		
		chr16: 86,505,840–86,506,078 (FC 1.0)*		

All depicted DMRs were hypermethylated in ACD/MPV samples except for one DMR detected in the ACD-mut vs. control analysis (*), this DMR was hypomethylated in ACD-mut samples. Genomic coordinates are based on Chr16(GRCh38)

We analysed DNA methylation patterns in ACD-del samples. Although methylated regions were present in both ACD-del samples and controls, no significant differences were detected between the groups (Table 2, Fig. 2b). Since one of the ACD-del patients (ACD-del3) harboured a deletion starting in the middle of the 60 kb enhancer, ACD-del samples ACD-del1 and ACD-del2 were also separately compared with control samples but again revealed no significant DMRs. These results indicate that the large maternal deletions in ACD-del samples have no effect on methylation patterns in the 60 kb *FOXF1* enhancer. Szafranski and colleagues previously suggested that the 60 kb *FOXF1* enhancer is subjected to allele specific imprinting through DNA methylation at the paternal allele [4]. However, they did not study allele specific methylation and therefore could not exclude allele specific imprinting through DNA methylation at the maternal allele. If the 60 kb *FOXF1* enhancer normally contains regions that are specifically methylated on the maternal allele, these would be lost in the ACD-del samples studied by us, resulting in significant DMRs between ACD-del and control samples (Additional file 5: Figure S 3A, left panel). Therefore, the absence of significant DMRs between ACD-del and control samples confirms that the 60 kb *FOXF1* enhancer is devoid of allele specific methylation on the maternal allele. Consequently, this means that if this *FOXF1* enhancer is subjected to allele specific imprinting through DNA methylation, this concerns the paternal allele (Additional file 5: Figure  S3A, right panel).

Using bisulphite sequencing, Schulze and colleagues recently identified a paternally imprinted region in the 60 kb *FOXF1* enhancer 10 kb upstream of LINC01082 (Hg38 chr16: 86,186,428–86,186,443) [16]. Since our ACD-del and control samples carried an intact paternal allele, we investigated whether this region was methylated in our samples. We did not detect abundant CpG methylation in this region, even though MeD-seq would be able to detect methylation at half of the CpG sites in this region if methylated, questioning the validity of paternal imprinting in this region (Additional file 5: Figure S 3B).

### Two hypermethylated regions in the 60 kb FOXF1 enhancer of ACD/MPV patients with FOXF1 point mutations

**Fig. 3:**
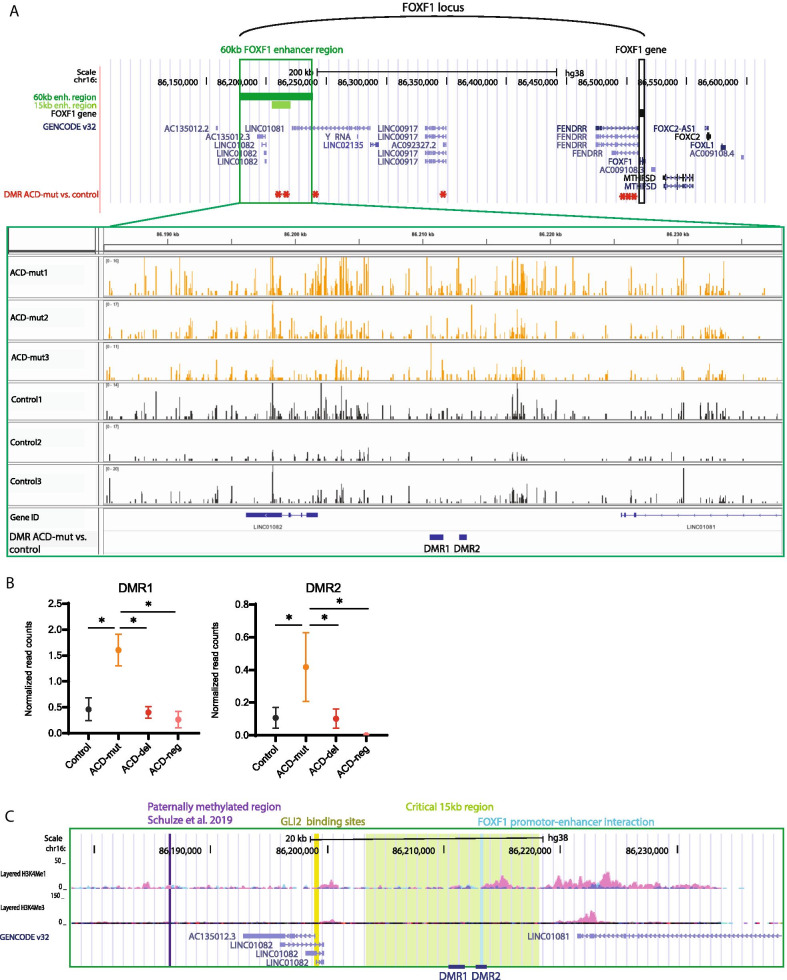
ACD-mut samples contain two hypermethylated regions in the 60 kb *FOXF1* enhancer potentially altering *FOXF1* expression. **a** Overview of the *FOXF1* locus in UCSC genome browser (GRCh38) and methylation patterns of ACD-mut samples (orange) and control samples (grey) in the 60 kb enhancer in IGV viewer. Red asterisk in upper panel: significant DMRs between ACD-mut and control samples. **b** Mean and standard deviation of normalized read counts in DMR1 and DMR2 for all lung samples (*p < 0.05 in group vs. group analysis).**c** Overview of the 60 kb enhancer region in UCSC genome browser (GRCh38). DMR1 and DMR2 are located within the 15 kb critical region (light green). DMR2 overlaps with the region that physically interacts with the *FOXF1* promotor (light blue) [10] and is located close to the H3K4Me1 peak of human lung fibroblasts (pink). Both DMRs are downstream of the previous proposed paternally methylated region (purple) [16] and GLI2 binding sites (yellow) [10]

Next, we compared methylation patterns between ACD-mut and control samples and found seven DMRs distributed over the *FOXF1* enhancer and the 250 kb region between the enhancer and *FOXF1* (Table 2; Fig. 3a). The two DMRs located inside the 60 kb *FOXF1* enhancer were both hypermethylated in ACD-mut samples compared to controls, but also compared to ACD-del and ACD-none samples (Fig. 3a, b). Interestingly, both DMRs were located within the 15 kb of the enhancer region which is thought to be the critical region that needs to be deleted in order to cause the typical ACD/MPV phenotype (Fig. 3c)[6]. Moreover, one of these DMRs (DMR2) encompasses the 250 bp that have been shown to physically interact with the *FOXF1* promoter [10]. Since ACD-mut samples do not contain single nucleotide polymorphisms (SNPs) in the identified DMRs, it is unclear whether only one or both parental alleles of ACD-mut samples are hypermethylated.

### Hypermethylation of the first FOXF1 exon in lung tissue of an ACD/MPV patient without a genomic FOXF1 variant

**Fig. 4:**
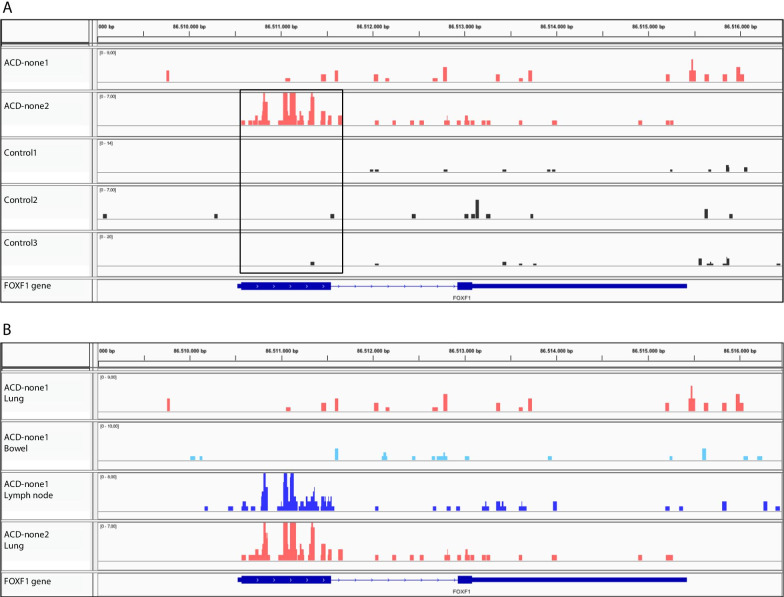
Hypermethylation of the first exon of *FOXF1* in an ACD/MPV patient without a genomic *FOXF1* variant **a** Hypermethylated region encompassing the first exon of *FOXF1* in ACD-none2, demonstrated in IGV viewer. Methylation patterns of ACD-none (red) and control samples (grey) are shown. **b** DNA methylation patterns of ACD-none lung tissues (light red), bowel tissue of ACD-none1 (light blue) and lymph tissue of ACD-none1 (dark blue) depicted with IGV viewer. Both lymph tissue of ACD-none1 and lung tissue of ACD-none2 demonstrate a highly methylated region covering exon 1

In our previous study, we described four patients with typical severe ACD/MPV phenotypes in which we could not detect genomic *FOXF1* alterations classified as pathogenic or likely pathogenic [3]. To investigate whether aberrant DNA methylation could contribute to the phenotypes of these patients, we compared methylation patterns of the two patients from which we had lung tissue available (ACD-none) with methylation patterns of control lung samples (Table 2). As we only had two samples of this group, normalized read counts in the *FOXF1* locus were manually reviewed without bioinformatical analysis. In contrast to the ACD-mut samples, we did not observe DMRs located in the *FOXF1* enhancer region. However, we found a highly methylated region in sample ACD-none2 that covered exon 1 of *FOXF1* (Fig. 4a). This hypermethylated region was not observed in any of the other lung samples tested in this study. Finally, since we had bowel and lymph node tissue of patient ACD-none1 available from autopsy, we compared the methylation pattern at the *FOXF1* locus in bowel, lymph node and lung tissues to explore the presence of tissue specific methylation patterns in the *FOXF1* locus. The DNA methylation patterns in bowel tissue showed no evident differences compared to the methylation pattern in lung tissue. However, lymph node tissue of ACD-none1 contained a highly methylated region covering exon 1 that was not present in lung tissue of ACD-none1 (Fig. 4b). As it was remarkable that this region was similarly methylated in the lung sample of ACD-none2 and not in any of the other lung tissues tested, the origin of each tissue block was re-confirmed by the department of pathology at the Erasmus MC.

## Discussion

The aim of the present study was to identify genome wide methylation patterns in lung tissue of ACD/MPV patients, with specific focus on the *FOXF1* locus to identify potential differences that might play a role in the pathogenesis of ACD/MPV. Comparison of ACD/MPV lung tissues with control lung tissues resulted in 319 DMRs genome wide, possibly affecting gene regulation of 115 protein coding genes. Interestingly, pathway analysis showed that the potentially upregulated genes are mainly involved in developmental processes. This conforms the developmental delay that is indicated by histological aspects of ACD/MPV lungs, such as reduced numbers of alveolar capillaries and increased numbers of type 2 pneumocytes [2]. For example, the most significantly enriched gene cluster of potentially upregulated genes included *TIE1* and *NOTCH1*, which are implicated in vascular development [27–32]. Furthermore, this cluster included *COL4A2* and *TGFB1*, which are involved in alveolar and airway branching morphogenesis respectively [33, 34]. *SOCS3*, an inhibitor of the JAK/STAT signalling pathway [35, 36] was among the potentially upregulated genes as well, in line with the previously demonstrated reduction of STAT3 protein in lung tissue of ACD/MPV patients [37]. The most significantly enriched gene cluster of potentially downregulated genes included *MUC5AC, ADAMTS2* and *GALNT15* which are all involved in O-linked glycosylation, a post-translational process regulating protein function. Of these, MUC5AC is secreted by goblet cells of the lower respiratory tract where it contributes to the mucocilliary clearance as part of the innate immune system [38–40]. However, the relevance of downregulation of O-linked glycosylation in relation to ACD/MPV is unclear. In summary, the results of our genome-wide methylation analysis implicate up- and downregulation of a range of genes in ACD/MPV. Whether these genes are indeed dysregulated and what their specific role is in ACD/MPV, requires further investigation. Additionally, similar to the patients included in the current study, genetic testing of ACD/MPV patients has been mainly directed at the *FOXF1* locus, potentially obscuring detection of alterations in other genes. Therefore, our findings could serve as a basis to expand genetic testing in ACD/MPV patients to study whether these genes harbour genomic alterations that contribute to ACD/MPV.

Through comparison of all included ACD/MPV lung samples together with control samples we did not find significant DMRs in the *FOXF1* locus. Similarly, no significant DMRs were found in the *FOXF1* locus when we compared the group of ACD-del samples with control samples. The three ACD-del samples included in this study harbour a large deletion (partly) encompassing the *FOXF1* enhancer on the maternal allele. And thus, the absence of DMRs in comparison indicates that the methylation pattern of the unaffected paternal allele is similar to the combined methylation patterns of both alleles in control samples. In line with previous studies, these results indicate that if the *FOXF1* enhancer is imprinted through DNA methylation, this involves the paternal allele (Additional file 4: Figure S 2A, right panel) [4, 6, 9, 16]. Although MeD-seq revealed multiple regions within the 60 kb enhancer that are methylated on the paternal allele of ACD-del samples, additional methylation studies and analyses are needed to define if these regions are paternally imprinted. For instance, a detailed SNP analysis on methylation data from multiple control samples could indicate whether certain regions at the 60 kb enhancer favour methylation at one allele. Using parental SNP data, one could identify on which parental allele the specific methylation occurs. Furthermore, bisulphite sequencing could be applied to identify paternally methylated regions using ACD-del samples. Since this technique also sequences unmethylated regions, it is possible to perform a quantitative comparison between the methylation in ACD-del samples harbouring a maternal deletion, and control samples. If specific regions in the 60 kb *FOXF1* enhancer are paternally imprinted, then 100% of the reads of the ACD-del samples would be methylated, compared to 50% of the reads in control samples. Due to DNA degradation in the FFPE tissue blocks that have been stored for many years, we were unable to perform bisulphite sequencing with the current collected samples [41].

When we compared the three ACD-mut samples with control samples, two significant hypermethylated regions were detected in the *FOXF1* enhancer, potentially interfering with normal gene regulation. Recently, it was shown that rare genomic variants in transcription factor binding sites can influence DNA methylation patterns, presumably leading to altered expression of nearby genes [42]. Although the mechanisms are unclear yet, it has been proposed that mutations could cause altered transcription factor binding, leading to formation of protein complexes with the DNA methylation machinery and thereby, alter DNA methylation. To investigate whether *FOXF1* point mutations influence methylation at the *FOXF1* enhancer, it would be interesting to study the DNA methylation status in primary human lung cells before and after introduction of a site-specific mutation in the *FOXF1* gene. Endothelial colony forming cells, for instance, have a high FOXF1 expression, are easily isolated from lung tissue, and may therefore, be a suitable cell type to investigate their methylation profile in the 60 kb enhancer before and after introduction of a *FOXF1* mutation[43]. However, it would be ideal to study multiple cell types so that cell type specific differences in methylation patterns can be recognized. One of the mechanisms by which *FOXF1* enhancer methylation could interfere with *FOXF1* regulation is by changing the interaction between the enhancer and the *FOXF1* promoter region. Therefore, it would be an interesting next step to use the ACD-mut samples to perform assay for transposase-accessible chromatin with sequencing (ATAC-Seq) [44] or universal NicE-seq (UniNicE-seq) [45]. This could clarify whether enhancer methylation alters the chromatin state in the TAD harbouring the *FOXF1* enhancer and gene, leading to an altered interaction.

In one of the ACD-none samples (ACD-none2) we detected a highly methylated region completely overlapping exon 1 of *FOXF1.* According to the Eukaryotic Promoter Database (EPD), the *FOXF1* core-promoter sequence ends approximately 40 bp downstream of the transcription start site (TSS) and 30 bp upstream of the start of exon 1 which means that the hypermethylated region does not overlap with the *FOXF1* promoter [46]. Currently, very little is known about the exact consequences of DNA methylation of exon 1 methylation on gene regulation. Therefore, it should be further investigated whether this hypermethylated region is associated with gene activation similar to other methylated regions overlapping gene bodies, or if this methylated region is close enough to the promoter to cause gene silencing, for instance by disrupting polymerase binding. Although only one lymph node sample was tested, the observation that exon 1 is hypermethylated in lymph tissue but not in the gastro-intestinal tissue or any of the lung tissues samples other than ACD-none2, could indicate that exon 1 methylation is indeed associated with reduced *FOXF1* expression since lymph node tissue is also the only tested tissue that normally does not express FOXF1 [47]. However, Szafranski and colleagues also found methylation of exon 1 in a normal lung sample [10]. Therefore, expression analyses in relation to exon 1 methylation are necessary before any conclusions about the relevance of the hypermethylated region in ACD-none2 can be drawn. In our previous study we showed that patient ACD-none2 carried a duplication in the 3’ UTR of *FOXF1* that was also present in the healthy father of the patient [3]. Although this variant has been classified as likely benign according to the ACMG classification [48], we cannot exclude that this variant contributed to the ACD/MPV phenotype, for instance through altering DNA methylation as suggested by our findings in ACD-mut samples. As multiple ACD/MPV patients have been described without a known genomic *FOXF1* variant, it would be interesting to investigate their methylation status to get a better indication of the contribution of abnormal *FOXF1* methylation in the pathogenesis of ACD/MPV [4].

In this study we investigated methylation patterns that led to many new hypotheses about the role of DNA methylation in the pathogenesis of ACD/MPV. To fully comprehend these results, additional expression analysis would be required. However, a limitation of this study was the quality of FFPE tissue blocks which was poor and highly variable, complicating reliable expression analyses. In our experience, these type of blocks frequently have partially degraded RNA, hampering RNA in situ hybridisations or qPCR analyses. Additional studies with FFPE blocks that have been processed similarly and stored for less than 5 years, or with fresh or frozen lung tissue, are needed to confirm our hypotheses.

Because DNA methylation is tissue specific, we used lung tissues to study methylation patterns that could contribute to the lung abnormalities observed in ACD/MPV patients [15, 21]. However, lung tissue contains a heterogeneous cell population and thus, the DMRs identified are not cell type specific. In human and mice lung tissue, FOXF1 is mainly expressed in mesenchymal, smooth muscle and endothelial cells [47, 49–51]. To study whether aberrant methylation of the *FOXF1* locus is specific for one of these cell types, it would be interesting to study DNA methylation patterns in sorted cells from fresh ACD/MPV lung tissues.

## Conclusion

This study provides genome wide methylation data on lung tissue of ACD/MPV patients. A detailed investigation of the *FOXF1* locus revealed hypermethylation of the *FOXF1* enhancer in ACD/MPV patients harbouring a point mutation in the *FOXF1* gene and abnormal hypermethylation of exon 1 in a patient without a genomic *FOXF1* variant. These abnormal methylation patterns potentially change the regulation of *FOXF1* contributing to the pathogenesis of ACD/MPV. This is the first study presenting *FOXF1* specific methylation data in relation to different genomic *FOXF1* variants associated with ACD/MPV and serves as important starting point for further research.

## Methods

### Sample collection

ACD/MPV and control FFPE lung tissue samples were collected from the Erasmus MC Sophia Children’s Hospital in Rotterdam, the VU University Medical Centre in Amsterdam and the Hospital for Sick Children in Toronto. All specimen were obtained as part of routine autopsy following informed consent of the parents/legal representatives or as part of a diagnostic procedure to diagnose ACD/MPV based on clinical suspicion. Samples were anonymized before they were subjected to MeD-seq. The research proposal was reviewed and approved by the Daily Board of the Medical Ethics Committee (METC) Erasmus University Medical Centre Rotterdam, The Netherlands.

### DNA isolation

DNA was isolated from FFPE lung tissue using the QIAamp DNA Mini Kit (Qiagen, Hilden, Germany), according to the manufacturer’s instructions. Eight to ten 10 µm sections were pre-treated with xylene to remove the paraffin and with sodium thiocyanate to permeabilize the tissue. Incubation with (ATL) lysis buffer and proteinase K was extended to 36 h to allow complete lysis. DNA concentrations were measured with the Quant-iT Picogreen assay kit (ThermoFisher Scientific) according the manufacturer’s instructions.

### MeD-seq sample preparation

DNA samples were prepared for MeD-seq as described previously [17]. In brief, DNA samples were digested with LpnPI (New England Biolabs, Ipswhich, MA, USA) and resulted in fragments of 32 bp with the methylated cytosine in the centre. Fragments were either purified on 10% TBE gel before preparation or purified by Pippin system gel after preparation. The 32 bp DNA fragments were prepared for sequencing using a ThruPlex DNA-seq 96D kit (Takara Bio Inc, Kusatasu, Japan) according to manufacturer’s protocol. To include dual indexed barcodes, stem–loop adaptors were blunt-end ligated to repaired input DNA and amplified (4 + 10 cycles) using a high-fidelity DNA polymerase. Multiplexed samples were sequenced on Illumina HiSeq2500 systems for single reads of 50 bp according to the manufacturer’s instructions. Dual indexed samples were demultiplexed using bcl2fastq software (Illumina).

### Data processing and analysis

MeD-seq data were processed and analysed with Python 2.7.5 using specifically created scripts as described previously [17]. In short, before mapping of the reads to the Hg38 genome using bowtie 2.1.0., the raw FASTQ files were subjected to Illumina adaptor trimming and filtered for the presence of LpnPI restriction sites 13-17 bp from the 3’ or 5’ end. For visualization of the mapped reads, BAM files were generated using SAMtools. LpnPI site scores were used to produce read count scores for the transcription start sites (TSS) [1 kb before and 1 kb after], gene bodies (1 kb after the TSS until the transcription end site) and CpG islands. Gene and CpG island annotations were downloaded from UCSC (hg38). To detect DMRs between two data sets, genome wide read counts were compared using the Chi-Squared test. Significance was set at *p* < 0.05 and was called with a Bonferroni correction or FDR using the Benjamini–Hochberg procedure. In addition, a genome-wide sliding window was used to detect sequentially differentially methylated LpnPI sites. Statistical significance was called between LpnPI sites in predetermined groups using the Chi-square test with a Bonferroni correction. Neighbouring significantly called LpnPI sites were binned and reported. Overlap of genome wide detected DMRs was reported for TSS, CpGisland or gene body regions using the annotations of UCSC (Hg38). Gene enrichment analyses were carried out using Metascape [26]. Metascape identifies significant enriched terms such as Gene Ontology (GO) terms and canonical pathways, and clusters them into a tree, based on Kappa-statistical similarities among their gene memberships [26].

## Supplementary Information

**Additional file 1**. **Table S1**. Lists of potentially up- and downregulated genes based on methylation status and TSS/GB overlap TSS: promotor, GB: Gene body. ↑: hypermethylated in ACD/MPV samples, ↓: hypomethylated in ACD/MPV samples.

**Additional file 2**. **Table S 2**. Results of gene ontology enrichment analysis using Metascape (26).

**Additional file 3**. **Figure S 1**. Simplistic illustration of the two models for parental imprinting of the 60kb FOXF1 enhancer Based on the suggested models by Szafranski et al. (4). Continuous arrow: full function of the FOXF1 enhancer. Dotted arrow: reduced function of the FOXF1 enhancer.

**Additional file 4**. **Figure S 2**. Haematoxylin and eosin staining of lung tissues of patients ACD-none1 and ACD-none2 demonstrating the main characteristics of ACD/MPV (2). Arrow: thickened alveolar wall. V: misaligned pulmonary vein. B: bronchiole. Scale bar: 200µm.

**Additional file 5**. **Figure S 3**. A: Models of maternal and paternal imprinting of the 60kb FOXF1 enhancers through DNA methylation. The absence of DMRs in ACD-del samples makes maternal imprinting of the FOXF1 enhancer unlikely and supports a paternal imprinting model. B: Methylation status of CpG sites of previously suggested paternally methylated region (rectangular frame) (16) in ACD-del and control samples. Read counts of each individual samples is demonstrated with IGV viewer (red: ACD-del, grey: control).

## Abbreviations

ACD/MPV: Alveolar capillary dysplasia with or without misalignment of the pulmonary veins
MeD-seq: Methylation DNA sequencing
FOXF1: Forkhead Box F1
CNV: Copy number variation
FFPE: Formalin-fixed and paraffin-embedded
DMR: Differentially methylated region
TSS: Transcription start site
