# Supplementary material for: Genome wide DNA methylation analysis of alveolar capillary dysplasia lung tissue reveals aberrant methylation of genes involved in development including the FOXF1 locus
Source: Clin Epigenetics. 2021 Jul 29;13:148. doi: 10.1186/s13148-021-01134-1 (PMC8323302; doi:10.1186/s13148-021-01134-1)
Supplement: Supplementary file 1 — Additional file 1. Table S1. Lists of potentially up- and downregulated genes based on methylation status and TSS/GB overlap TSS: promotor, GB: Gene body. ↑: hypermethylated in ACD/MPV samples, ↓: hypomethylated in ACD/MPV samples. [file 13148_2021_1134_MOESM1_ESM.docx]

Potentially upregulated genes

| **Gene ID** | **DMR overlap with TSS or GB** | **Methylation ACD/MPV** | **Fold Change** |
| --- | --- | --- | --- |
| TIE1 | GB | ↑ | 7.91 |
| RERE | GB | ↑ | 5.09 |
| SYN3 | GB | ↑ | 5.06 |
| TIMP3 | GB | ↑ | 5.06 |
| HOXB3 | GB | ↑ | 4.68 |
| HOXB6 | GB | ↑ | 4.68 |
| CIRBP | GB | ↑ | 4.61 |
| AMZ1 | GB | ↑ | 4.55 |
| GEMIN4 | GB | ↑ | 4.03 |
| HOXD3 | GB | ↑ | 4.00 |
| BAIAP2 | GB | ↑ | 3.91 |
| MIDN | GB | ↑ | 3.83 |
| VAC14 | GB | ↑ | 3.75 |
| PEBP4 | GB | ↑ | 3.72 |
| SOCS3 | GB | ↑ | 3.70 |
| KDM6B | GB | ↑ | 3.68 |
| RIMBP2 | GB | ↑ | 3.61 |
| ADAMTSL4 | GB | ↑ | 3.60 |
| SH3D21 | GB | ↑ | 3.43 |
| EVA1B | GB | ↑ | 3.43 |
| RAD51B | GB | ↑ | 3.43 |
| IRF8 | GB | ↑ | 3.38 |
| TBC1D1 | GB | ↑ | 3.36 |
| CCDC40 | GB | ↑ | 3.30 |
| HOXA3 | GB | ↑ | 3.28 |
| C6orf132 | GB | ↑ | 3.15 |
| DHRS3 | GB | ↑ | 3.12 |
| CHMP1A | GB | ↑ | 3.08 |
| CBX4 | GB | ↑ | 3.08 |
| ARRB1 | GB | ↑ | 3.07 |
| CYGB | GB | ↑ | 3.02 |
| PRCD | GB | ↑ | 3.02 |
| PDE11A | GB | ↑ | 3.00 |
| SART1 | GB | ↑ | 2.95 |
| PKNOX2 | GB | ↑ | 2.92 |
| PRKAR1B | GB | ↑ | 2.91 |
| ADAMTS17 | GB | ↑ | 2.86 |
| TAOK2 | GB | ↑ | 2.80 |
| CROCC | GB | ↑ | 2.76 |
| GATA2 | GB | ↑ | 2.75 |
| GPR133 | GB | ↑ | 2.75 |
| NXN | GB | ↑ | 2.72 |
| PTBP1 | GB | ↑ | 2.70 |
| RXRA | GB | ↑ | 2.69 |
| NOTCH1 | GB | ↑ | 2.69 |
| SNX33 | GB | ↑ | 2.67 |
| CACNA1H | GB | ↑ | 2.66 |
| COL23A1 | GB | ↑ | 2.63 |
| TMTC2 | GB | ↑ | 2.58 |
| NFIC | GB | ↑ | 2.57 |
| ERICH1 | GB | ↑ | 2.56 |
| SIPA1 | GB | ↑ | 2.48 |
| PCGF3 | GB | ↑ | 2.46 |
| PRKCZ | GB | ↑ | 2.45 |
| SEPT9 | GB | ↑ | 2.42 |
| DENND3 | GB | ↑ | 2.41 |
| SPTB | GB | ↑ | 2.40 |
| MRPS9 | GB | ↑ | 2.38 |
| TGFB1 | GB | ↑ | 2.29 |
| CCDC97 | GB | ↑ | 2.29 |
| COL4A2 | GB | ↑ | 2.28 |
| BAZ2A | GB | ↑ | 2.27 |
| RECQL5 | GB | ↑ | 2.22 |
| FAM110A | GB | ↑ | 2.20 |
| SLC16A3 | GB | ↑ | 2.16 |
| CSNK1D | GB | ↑ | 2.16 |
| RPTOR | GB | ↑ | 2.15 |
| KATNAL2 | GB | ↑ | 2.15 |
| TCEB3CL | GB | ↑ | 2.15 |
| RAB12 | GB | ↑ | 2.08 |
| CHST15 | GB | ↑ | 2.08 |
| KIFC3 | GB | ↑ | 2.08 |
| LRFN5 | GB | ↑ | 2.07 |
| SEC16A | GB | ↑ | 2.06 |
| TENM4 | GB | ↑ | 2.04 |
| KRTAP10-3 | TSS | ↓ | 2.04 |
| PRDM16 | GB | ↑ | 2.02 |
| EHMT1 | GB | ↑ | 2.01 |
| ABCC2 | GB | ↑ | 2.01 |

Potentially downregulated genes

| **Gene ID** | **DMR overlap with TSS or GB** | **Methylation ACD/MPV** | **Fold change** |
| --- | --- | --- | --- |
| PLEC | TSS | ↑ | 7.11 |
| GALNT15 | GB | ↓ | 5.65 |
| CYP2E1 | GB | ↓ | 5.46 |
| DPEP1 | GB | ↓ | 4.28 |
| LBX2 | TSS | ↑ | 3.82 |
| ADARB2 | GB | ↓ | 3.55 |
| ZNF835 | GB | ↓ | 3.44 |
| AK1 | TSS | ↑ | 3.29 |
| CCDC6 | GB | ↓ | 3.08 |
| LSG1 | GB | ↓ | 3.01 |
| RBFOX1 | GB | ↓ | 2.88 |
| SUGCT | GB | ↓ | 2.65 |
| PDE11A | GB | ↓ | 2.60 |
| POLRMT | GB | ↓ | 2.60 |
| TOLLIP | GB | ↓ | 2.52 |
| SMYD3 | GB | ↓ | 2.47 |
| ADAMTS2 | GB | ↓ | 2.46 |
| PTH2R | GB | ↓ | 2.46 |
| EFTUD1 | GB | ↓ | 2.42 |
| PCBP3 | GB | ↓ | 2.42 |
| DLGAP2 | GB | ↓ | 2.33 |
| TULP4 | GB | ↓ | 2.33 |
| PIGQ | GB | ↓ | 2.27 |
| ERICH1 | GB | ↓ | 2.25 |
| IL1A | GB | ↓ | 2.24 |
| C10orf76 | GB | ↓ | 2.21 |
| PTPRN2 | GB | ↓ | 2.21 |
| CCDC47 | GB | ↓ | 2.18 |
| DIP2C | GB | ↓ | 2.18 |
| MUC5AC | GB | ↓ | 2.16 |
| PRR21 | TSS | ↑ | 2.12 |
| KLHDC4 | GB | ↓ | 2.12 |
| TTC34 | GB | ↓ | 2.11 |
| MFSD8 | GB | ↓ | 2.07 |
| INPP5E | TSS | ↑ | 2.06 |
| TSPEAR | GB | ↓ | 2.04 |
