# Supplementary figures and images for: Genome wide DNA methylation analysis of alveolar capillary dysplasia lung tissue reveals aberrant methylation of genes involved in development including the FOXF1 locus
Source: Clin Epigenetics. 2021 Jul 29;13:148. doi: 10.1186/s13148-021-01134-1 (PMC8323302; doi:10.1186/s13148-021-01134-1)

A

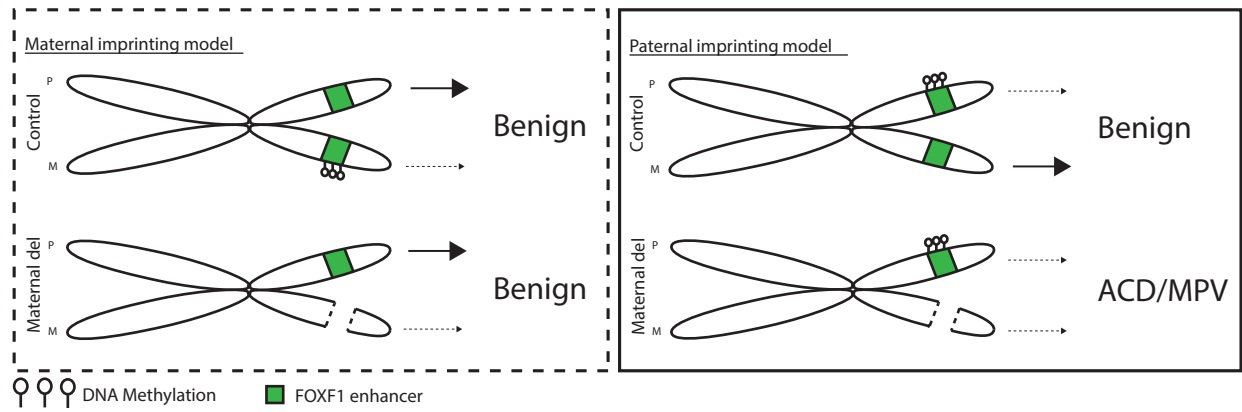

Supplement: Supplementary file 3 — Additional file 3. Figure S 1. Simplistic illustration of the two models for parental imprinting of the 60kb FOXF1 enhancer Based on the suggested models by Szafranski et al. (4). Continuous arrow: full function of the FOXF1 enhancer. Dotted arrow: reduced function of the FOXF1 enhancer. [file 13148_2021_1134_MOESM3_ESM.pdf]

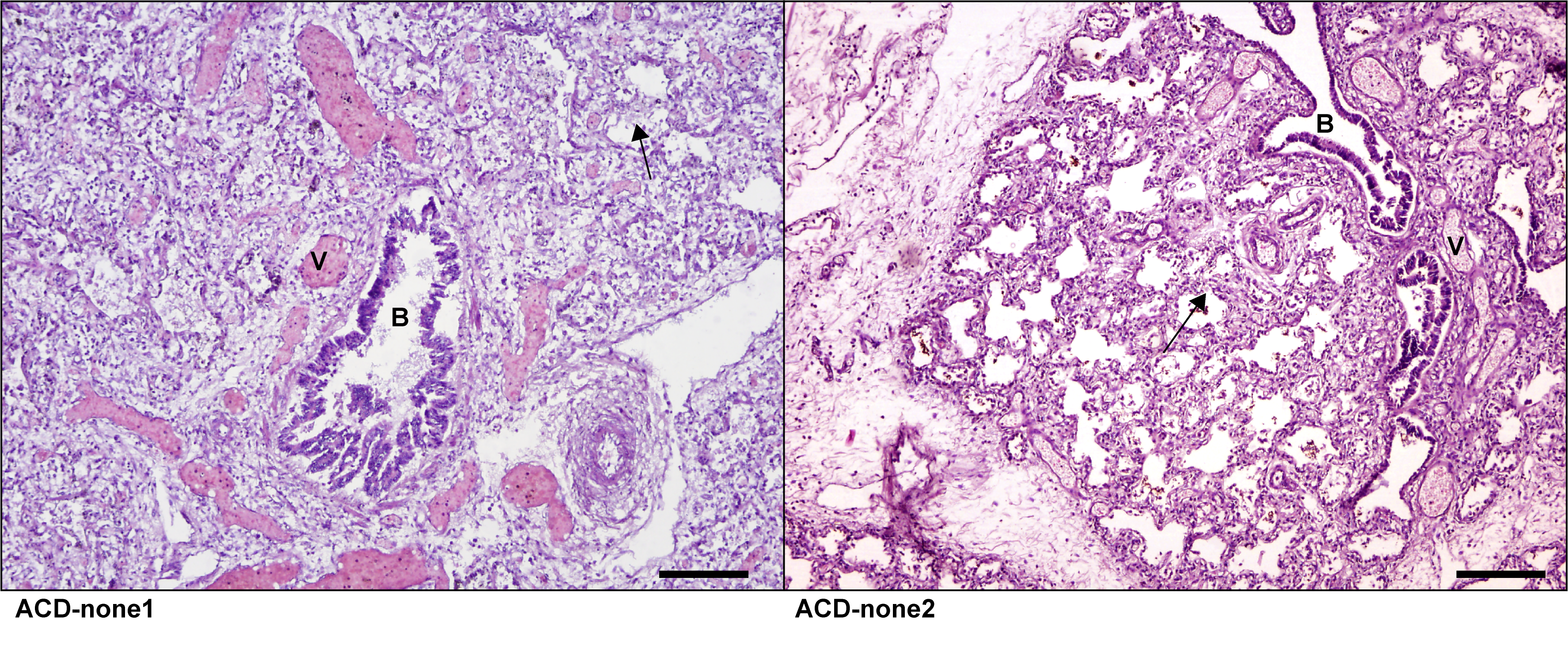

Supplement: Supplementary file 4 — Additional file 4. Figure S 2. Haematoxylin and eosin staining of lung tissues of patients ACD-none1 and ACD-none2 demonstrating the main characteristics of ACD/MPV (2). Arrow: thickened alveolar wall. V: misaligned pulmonary vein. B: bronchiole. Scale bar: 200µm. [file 13148_2021_1134_MOESM4_ESM.tif]

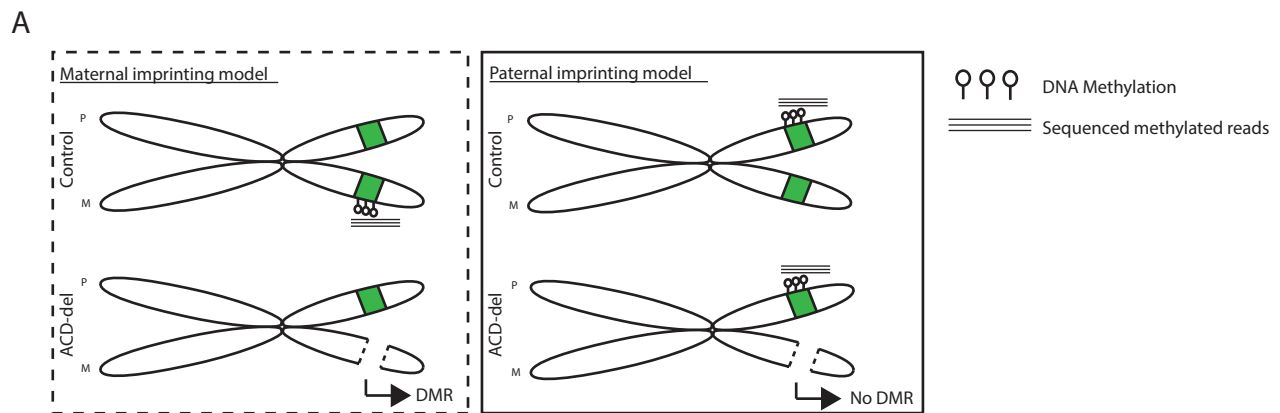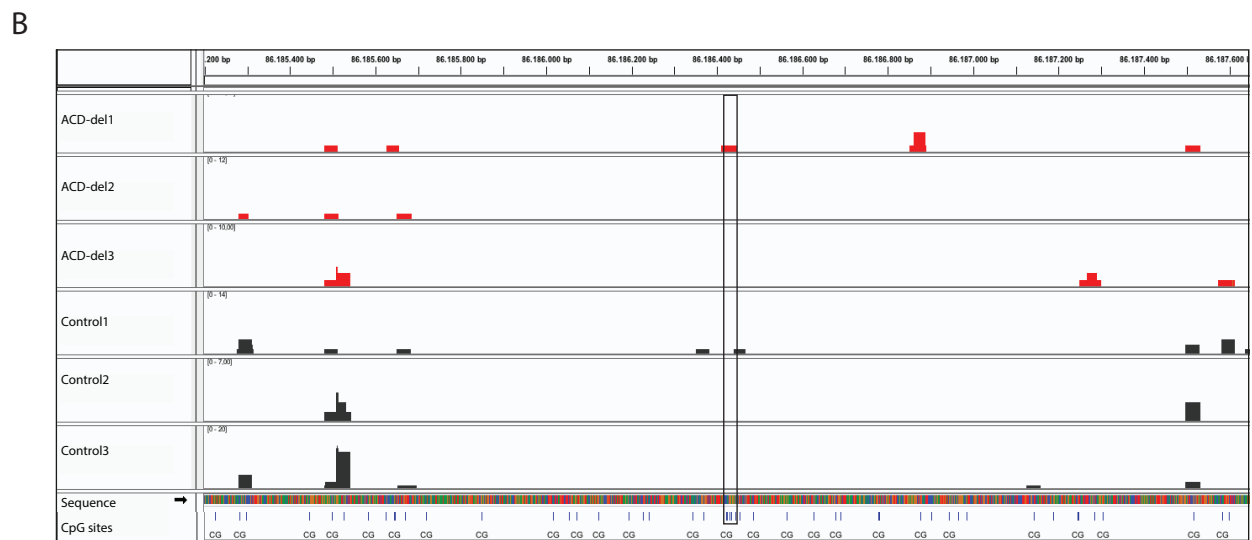

Supplement: Supplementary file 5 — Additional file 5. Figure S 3. A: Models of maternal and paternal imprinting of the 60kb FOXF1 enhancers through DNA methylation. The absence of DMRs in ACD-del samples makes maternal imprinting of the FOXF1 enhancer unlikely and supports a paternal imprinting model. B: Methylation status of CpG sites of previously suggested paternally methylated region (rectangular frame) (16) in ACD-del and control samples. Read counts of each individual samples is demonstrated with IGV viewer (red: ACD-del, grey: control). [file 13148_2021_1134_MOESM5_ESM.pdf]
